# Identification of Koala (*Phascolarctos cinereus*) Faecal Cortisol Metabolites Using Liquid Chromatography-Mass Spectrometry and Enzyme Immunoassays

**DOI:** 10.3390/metabo11060393

**Published:** 2021-06-16

**Authors:** Flavia Santamaria, Christopher K. Barlow, Rolf Schlagloth, Ralf B. Schittenhelm, Rupert Palme, Joerg Henning

**Affiliations:** 1 Flora, Fauna and Freshwater Research and Koala Research Central Queensland, School of Health, Medical and Applied Sciences, Central Queensland University, North Rockhampton, QLD 4702, Australia; r.schlagloth@cqu.edu.au; 2 Monash Proteomics and Metabolomics Facility, Department of Biochemistry and Molecular Biology, Monash University, Clayton, VIC 3800, Australia; chris.barlow@monash.edu (C.K.B.); ralf.schittenhelm@monash.edu (R.B.S.); 3 Department of Biomedical Sciences, University of Veterinary Medicine, 1210 Vienna, Austria; 4 School of Veterinary Science, The University of Queensland, Gatton, QLD 4343, Australia; j.henning@uq.edu.au

**Keywords:** *Phascolarctos cinereus*, koala, non-invasive, stress, faecal glucocorticoid metabolites, LC-MS, HPLC

## Abstract

The koala (*Phascolarctos cinereus*) is an arboreal folivorous marsupial endemic to Australia. Anthropogenic activities and climate change are threats to this species’ survival and are potential stressors. A suitable non-invasive method is needed to objectively detect stress in koalas. Under conditions of stress, the concentration of the hormone cortisol in plasma or in saliva is elevated, and this would provide a convenient measure; however, collecting blood or saliva from wild animals is both practically difficult and stressful, and so likely to confound any measurement. In contrast, measurement of cortisol metabolites in faeces provides a practical and non-invasive method to objectively measure stress in koalas. Unfortunately, the identity of the main faecal cortisol metabolites of koalas is unknown. In this study, we have used both untargeted liquid chromatography–mass spectrometry (LC-MS) and enzyme immunoassays (EIAs) to identify several faecal cortisol metabolites in two koalas, one female (18 months old, 4.1 kg) and one male (4 years old, 6.95 kg) upon administration of hydrocortisone (cortisol) sodium succinate. The LC-MS analysis identified tetrahydrocortisol along with several other isomers as cortisol metabolites. After a survey of five enzyme immunoassays, we found that two metabolites, tetrahydrocortisol and 3β-allotetrahydrocortisol, could be detected by EIAs that used antibodies that were raised against their structurally similar corticosterone counterparts, tetrahydrocorticosterone and 3β-allotetrahydrocorticosterone, respectively. While the 3β-allotetrahydrocortisol metabolite was detected in the faeces of only one of the two animals studied, tetrahydrocortisol was detected in both. These results ultimately indicate that tetrahydrocortisol is likely the main faecal cortisol metabolite in koalas, and we demonstrate that it can be measured by an EIA (50c) that was originally developed to measure tetrahydrocorticosterone.

## 1. Introduction

The koala (*Phascolarctos cinereus*) is an arboreal folivore marsupial mammal, endemic to Australia. Koalas are found along eastern and south-eastern Australia in the states of Queensland, New South Wales, and Victoria as well as in the Australian Capital Territory and a small area of South Australia. Unfortunately, the distribution and density of this species is declining, which has led to the listing of koala populations in Queensland, New South Wales, and in the Australian Capital Territory as vulnerable under national and state environment laws [1]. Loss and fragmentation of habitat, anthropogenic threats linked to urbanisation, such as collisions with cars and attacks by dogs, as well as changing climatic conditions, for instance more frequent, severe, and prolonged droughts, are major stressors for koalas and are threats to the survival of this species [2]. A non-invasive method is needed for researchers, veterinarians, and conservation managers to confidently detect acute and chronic stress in koalas and to understand the impact of stress on morbidity and mortality events in koalas.

Glucocorticoids (GCs), cortisol and corticosterone, are steroid hormones that regulate several metabolic and physiological processes [3] and are involved in the response to various stressors [4,5,6,7,8]. Studies on stress metabolism in marsupials, including koalas [9,10,11,12], indicated that the main stress hormone in these mammals is cortisol. Measurement of GCs in blood, from animals in the wild, involves their capture and subsequent handling, which, in addition to being practically challenging, is stressful and therefore likely to confound GCs measurements. In contrast, collection of faecal samples is both readily achievable and non-invasive. In most species, however, GCs are almost completely metabolised prior to excretion with only trace quantities, if any, of the parent hormone remaining [5,13,14]. Several studies have demonstrated that increased plasma GC concentration corresponds to a subsequent increase in faecal cortisol metabolites (FCMs) in many species, including koalas [14,15,16,17]. Consequently, the measurement of FCMs is frequently utilized as an indication of stress [18].

The metabolism of cortisol prior to excretion is typically complex. In many mammalian species, cortisol undergoes a series of irreversible enzymatic reactions that produce 5α- and 5β-tetrahydrocortisol isomers in the liver. These metabolites may be further conjugated with sulphate or glucuronides, increasing their solubility and allowing their excretion in urine via the kidney or faeces via the intestine [19,20,21]. Additionally, bacterial species in the intestinal tract are capable of side-chain cleavage of cortisol metabolites as shown in humans [22] and in sheep and cow [13] and cause further degradation of FCMs [23]. Due to this metabolomic complexity, the FCM profile may vary substantially between species and so present an analytical challenge to faithfully identify the main FCMs for a particular species.

Identification of FCMs is best achieved by administration of radiolabelled cortisol, either ^3^H or ^14^C, to increase cortisol in circulation and determine its metabolic pathway and metabolism in mammalian species [21,24,25,26]. The use of radiolabelled tracers is expensive and presents additional complications associated with exposure to radioactivity. An alternative strategy is to avoid radiolabelling altogether and to try to identify FCMs by untargeted liquid chromatography-mass spectrometry (LC-MS). Here, we have intravenously injected a therapeutical dose [27] of hydrocortisone sodium succinate (HCSS) and identified koala FCMs using an untargeted LC-MS approach.

Once the main FCMs of a species are identified, two main analytical approaches have been used for their measurement: LC-MS and enzyme immunoassays (EIAs). LC-MS is typically highly specific and capable of measuring multiple molecular species in a single analysis [28,29]. It has seen limited application to FCMs however largely due to the cost and requirement for specialized instrumentation and expertise [14,18]. In contrast, EIAs are relatively inexpensive and straightforward to use. Unfortunately, the number of commercially available EIAs for the measurement of cortisol metabolites is limited and EIAs are prone to problems of cross-reactivity.

The problem of cross-reactivity in EIAs is particularly severe for small molecules where related metabolites may have almost identical structures and essentially share an epitope. Indeed, this cross-reactivity has been leveraged in many instances to provide a measure of FCMs without identification of those metabolites being measured. Under this strategy the antibody produced would ideally show a high degree of cross-reactivity across several FCMs, and Möstl et al. [30,31] have described “group” specific antibodies that explicitly aim to achieve this goal. Unfortunately, a large number of studies utilize commercially available EIAs targeting the parent GC that, in contrast to the group specific assays, are typically designed to minimise cross-reactivity [5]. While this approach is readily implemented, it has several obvious drawbacks. The metabolite(s) causing the response are unknown and may correspond to minor metabolomic products. In contrast, the most abundant FCMs may exhibit no cross-reactivity with the antibody being used and so escape detection completely. As the metabolites being measured are likely to have very different responses to the assays target, they can only provide relative quantitation, making comparisons with other EIAs and across studies very difficult. Consequently, for each EIA a thorough validation for each species is required to ensure that appropriate assays are selected to detect species-specific FCMs [5,14,17,21].

Recent studies in koalas [10,17,32] have used ACTH injections to increase plasma cortisol and validate a number of EIAs that may detect FCMs. These studies have typically reported comparatively low concentrations in koalas, which may reflect some of the analytical challenges associated with using cortisol EIAs. Here, we attempted to identify the major FCMs in koalas using complementary LC-MS and EIA analysis in order to provide a more robust basis for FCM detection.

## 2. Results

### 2.1. Effect of Blood Sampling and Injection

Despite some signs of displeasure during the blood sampling conducted prior to the HCSS injection and during the HCSS injection, the two koalas did not display any signs of stress. They acted, ate, and vocalised after the injection as they did during the 24 h before the procedure, and we did not detect any abnormal behaviour.

### 2.2. Untargeted LC-MS Analysis

**Figure 1:**
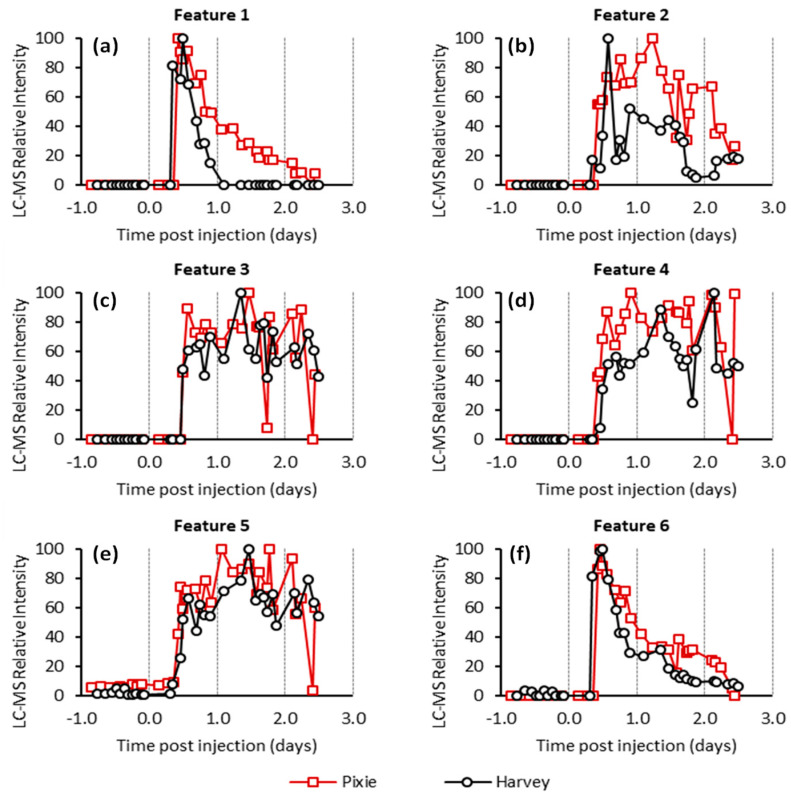
Relative intensities for features 1 to 6 (**a**–**f** respectively) from the untargeted LC-MS analysis. The intensities scaled to the most abundant measurement within the series.

Pellets were extracted and subjected to untargeted LC-MS analysis in an attempt to identify FCMs. To provide the broadest possible analysis, the mass spectrometer was operated in both positive and negative mode producing cationic and anionic ions, respectively. Analysis of the LC-MS data results in the identification of several thousand chromatographic features for each animal. In the absence of isotopic labelling, identifying which of these correspond to metabolites of cortisol presents a significant analytical challenge. Features corresponding to cortisol metabolites should be significantly more intense after cortisol administration (AC samples) in comparison to those collected before cortisol administration (BC samples). Accordingly, only features were selected that were substantially elevated in the AC samples compared to those of the BC samples (specifically, at least 10-fold greater than the highest BC samples). The time-course profile of each of these features was then scrutinized to exclude those features for which a single or small number of non-consecutive high values were observed as these are unlikely to correspond to true metabolites of cortisol, but rather chemical noise or inconsistent integration by the peak extraction algorithm. Ultimately, we focused on six features that were found to be elevated following cortisol administration in both animals (Table 1 and Figure 1).

**Figure 2:**
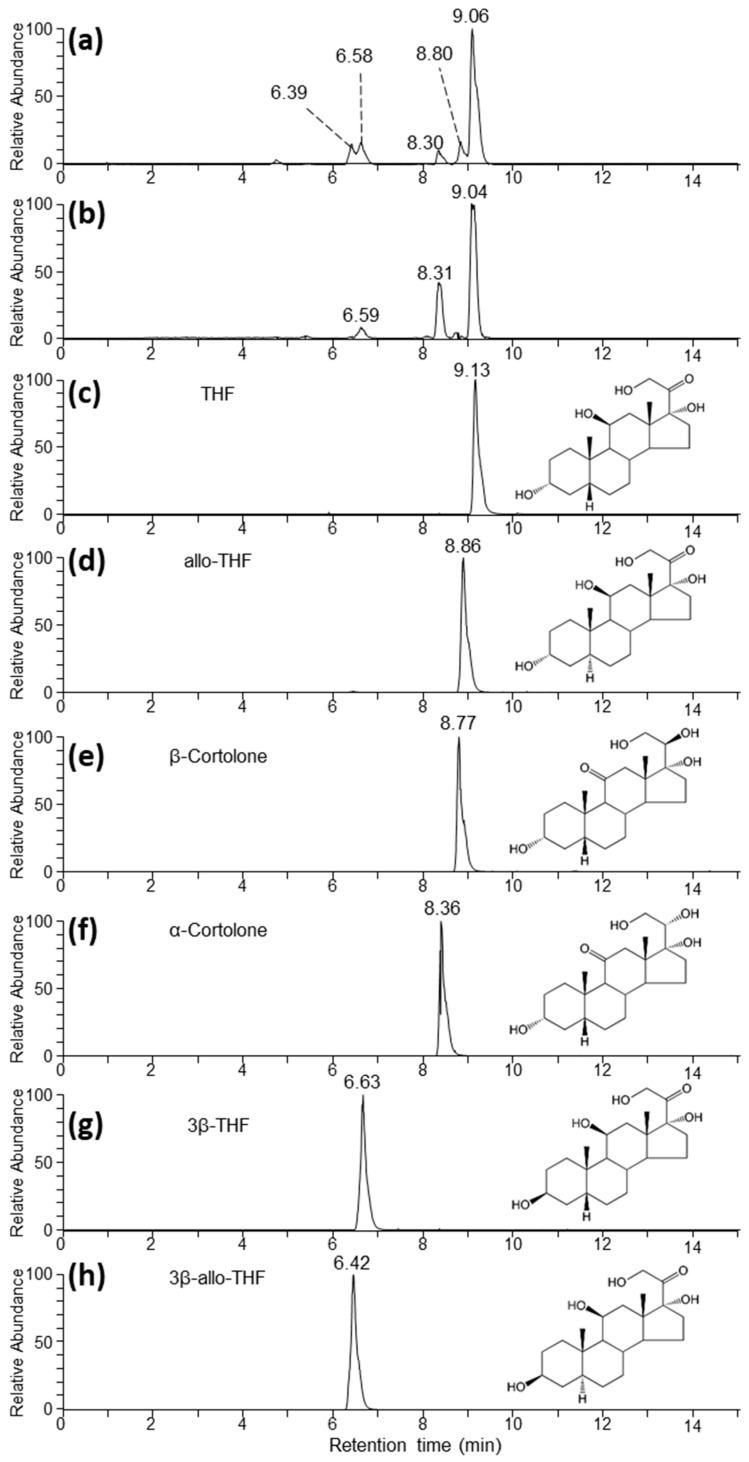
Extracted ion chromatograms (EICs) corresponding to the *m/z* of the formate adduct of THF, its isomers, and α- and β-cortolone under reverse phase chromatographic conditions. (**a**,**b**) correspond to representative AC samples from Harvey and Pixie, respectively, from timepoints that were high in feature 2 according to the untargeted analysis. (**c**–**h**) correspond to authentic standards as indicated.

Features 2 to 5 had similar kinetic profiles with low intensity or no detection in the first few AC samples followed by a broad profile across the remaining samples (Figure 2). Feature 2 had a neutral mass of 366.240 Da, consistent with the mass of tetrahydrocortisol (THF) and/or its isomers. Features 3, 4, and 5 appeared to have similar molecular formulas to feature 2, differing only by the addition of an oxygen, water, and loss of a methylene (CH_2_) unit, respectively. This suggests that they are likely to be structurally and metabolically related.

To facilitate the identification of the metabolite corresponding to feature 2 we undertook LC-MS analysis of authentic standards of THF and several of its isomers (Table 2). Analysis using the same HILIC chromatographic conditions used in the untargeted analysis resulted in poor retention of the standards, making distinction between them impossible (data not shown). In contrast, the use of a reverse phase chromatographic approach was more successful in separating the isomeric standards (Figure 2). Under these conditions we primarily detected THF and related isomers as formate adducts [M + CHO_2_^−^]^−^. Analysis of a subset of samples, which gave the greatest intensity for feature 2 from the two animals (Figure 2a,b), showed several peaks in the extracted ion chromatogram (EIC) corresponding to the THF formate adduct, suggesting that feature 2 may have corresponded to multiple co-eluting isomeric metabolites. Specifically, for both animals we observed a major peak at around 9.05 min, which compared favourably with the THF standard eluting at 9.13 min. Additionally, the feature at 8.31 min was a good match for the α-cortolone standard, which eluted at 8.36 min (Figure 2f). To confirm these assignments, the features around 9.1 and 8.3 min were further characterized by gas-phase fragmentation (MS/MS). As expected, the resulting spectra matched closely with the ones obtained from the THF and α-cortolone standards, respectively (Supplementary Materials Figure S1). Additionally, we observed a number of other chromatographic features around 6.4, 6.6, and 8.8 min, which aligned closely with 3β-allo-THF (Figure 2h), 3β-THF (Figure 2g), and allo-THF (Figure 2d). In these cases, there was insufficient signal to allow confirmation by MS/MS.

**Figure 3:**
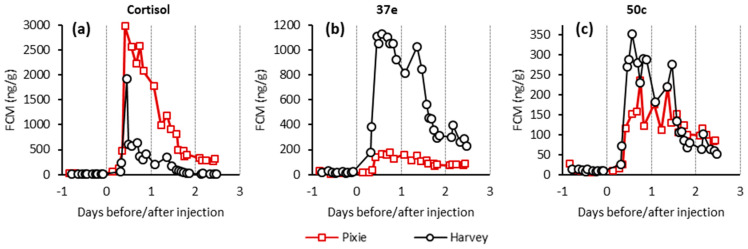
EIAs used for analyses of faecal extracts: (**a**) Cortisol EIA, (**b**) 37e EIA, and (**c**) 50c EIA. Red lines represent Pixie and black, Harvey.

Feature 1 had a neutral mass of 462.225 Da, consistent with the mass of hydrocortisone succinate. This assignment was supported by analysis under reverse phase chromatographic conditions in which we found good agreement between the retention of the extracted ion chromatogram corresponding the [M − H]^−^ anion of feature 1 and a hydrocortisone succinate standard (Supplementary Figure S2). The kinetic profile of this feature (Figure 1a) showed a maximum intensity shortly after cortisol administration followed by an almost complete decline by 1 and 2 days in the case of the Harvey and Pixie, respectively. This profile was highly correlated with the concentration of cortisol as measured using the cortisol EIA (Figure 3a).

Feature 6 also displayed a similar kinetic profile to feature 1. The mass of this feature was consistent with the ammonium adduct of the succinate ester of THF or cortolone isomers (Supplementary Materials Figure S3a,b). Inspection of the data collected using reverse phase chromatography revealed multiple chromatographic features in the AC (Supplementary Materials Figure S3c,d), but not the BC samples. Specifically, the most intense feature was observed at 7.6 min with a number of smaller features at 4.6, 5.6, 6.8, and 7.4 min. This was reminiscent of the pattern observed for the THF and related isomers Figure 2a,b and it seemed probable that the features here correspond to the succinate ester derivatives of those metabolites.

### 2.3. Enzyme Immunoassay

In addition to LC-MS, the samples were analysed by five EIAs, described in the Materials and Methods section (Table 4). Of these assays, cortisol, 37e, and 50c EIAs showed profiles with substantially elevated signals following cortisol administration (Figure 3) while 69a and 72T did not (Supplementary Materials Figure S4). The highest values detected with the cortisol EIA was 2979 ng/g after 11 h following IV injection for the female and 1922 ng/g after 10 h for the male. The cross-reactivity of these assays was tested against THF and related isomers (Table 3). The cortisol EIA showed substantial cross-reactivity against 3β-allo-THF (25%). The 37e EIA showed almost perfect cross-reactivity, 100% against 3β-allo-THF, which is perhaps unsurprising given the immunogen of that assay is its corticosterone counterpart, 3β-allo-tetrahydrocorticosterone, coupled at C20. Similarly, the 50c EIA that targets tetrahydrocorticosterone showed a high degree of cross-reactivity (20.7%) with its cortisol counterpart THF.

### 2.4. EIA and LC-MS Analysis of Fractionated Pooled Samples

**Figure 4:**
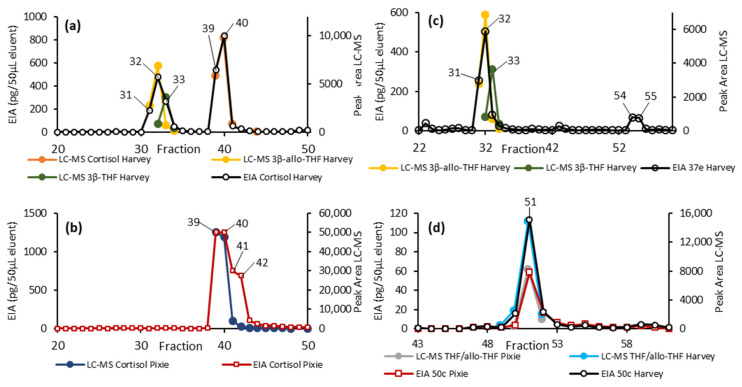
HPLC fractionation of the pooled AC samples from Harvey and Pixie. Here, we overlaid the detection by EIA (primary *y*-axis) and targeted LC-MS (secondary *y*-axis). (**a**,**b**) show the results from the cortisol EIA together with the LC-MS peak areas for the relevant metabolites for Harvey and Pixie, respectively. (**c**) shows the results of the 37e EIA for Harvey together with the LC-MS peak areas for 3β-allo-THF and 3β-THF. (**d**) overlays the results from the 50c EIA and LC-MS peak area for THF for both animals.

To determine the metabolites responsible for the EIA activity, several AC samples from each animal were pooled and then fractionated by HPLC. The resulting HPLC fractions were then tested against the cortisol, 37e, and 50c EIAs, and those fractions showing immunoreactivity were further analysed by LC-MS (Figure 4).

Fractions 31 to 33 from the male (Harvey) sample showed activity in both the cortisol and 37e EIAs. LC-MS analysis detected 3β-allo-THF in all three of these fractions and the closely related 3β-THF was detected in 32 and 33 (Figure 4a). Both the cortisol and 37e EIAs show significant cross-reactivity with 3β-allo-THF but not 3β-THF, and so the activity in these fractions can be attributed to 3β-allo-THF and not 3β-THF.

Fractions 54 and 55 from Harvey gave a response in the 37e EIA, although we were unable to identify the responsible metabolite. Fractions 39 to 41 from the male (Harvey) and 39 to 42 from the female (Pixie) showed substantial activity in the cortisol EIA. LC-MS analysis indicated that the cortisol predominately eluted in fractions 39 and 40 (Figure 4a,b) and the EIA and LC-MS responses across these fractions seemed well correlated. The EIA response in fractions 41 and 42 observed in the female (Pixie) were not readily accounted for by free cortisol, suggesting an additional metabolite may be responsible for the activity in those two fractions.

Fractions 50 to 52 showed substantial activity in the 50c EIA. LC-MS analysis found a substantial signal corresponding to THF and/or allo-THF, which in this case could not be distinguished chromatographically. The 50c EIA showed substantial cross-reactivity to THF, but negligible cross-reactivity towards allo-THF (Table 3). Taken together these results indicated the active species was THF.

## 3. Discussion

Habitat loss, urbanization, climate change, and wildfires are major stressors affecting koalas. Therefore, a species-specific [10,17] monitoring tool such as an EIA that could evaluate the stress response in koalas, by detecting the main glucocorticoid metabolites, needs to be developed. Thus, the intent of this study was to identify the most abundant FCMs found in koalas using untargeted LC-MS analysis and identify the best EIA able to detect them.

Untargeted LC-MS analysis resulted in the identification of six chromatographic features, which were substantially and consistently elevated in several faecal samples following injection with HCSS. Feature 1 corresponded to the parent drug. Feature 2 was consistent with THF and/or an isomeric metabolite. Further analysis by reverse phase chromatography demonstrated that feature 2 did indeed correspond to a mixture of several isomeric metabolites, of which THF was the most abundant. Feature 3, 4, and 5 remained unassigned but appear to have molecular formulas extremely similar to that of THF, suggesting that they are closely related metabolites. Finally, feature 6 appeared to correspond to several metabolites that may be the succinate esters of THF and related isomers and so, along with feature 1, may be considered an artifact of the HCSS administration.

Based on these results, THF appears to be the most prominent FCM detected. However, to the best of our knowledge, no EIA that specifically targets THF is available. Consequently, we tested several EIAs, of which cortisol, 37e, and 50c EIAs showed the highest increases in FCM values following HCSS injection. Subsequent fractionation and analysis by LC-MS of those fractions, giving a substantial response in the EIA, allowed further identification of the metabolite responsible. Although the cortisol EIA provided the greatest apparent concentration, this can largely be attributed to the presence of free cortisol, which we consider to be an artifact of the comparatively high dose of HCSS administered. The cortisol assay also detected 3β-allo-THF in the male samples, and there is evidence that it detected an additional, but yet unidentified, metabolite in the female samples in fractions 41 and 42. Similarly, the 37e EIA (Figure 4c) also detected 3β-allo-THF which was present in the male, but not in the female samples. The antibody used in the 37e EIA was originally raised against 3β-allotetrahydrocorticosterone, the corticosterone analogue of 3β-allo-THF (corticosterone differs from cortisol in lacking a 17α hydroxy). The 3β-allo-THF shows cross-reactivity in this assay, explaining earlier findings in other mammals where cortisol is the dominant GC hormone, and this EIA proved well suited for measuring FCMs [33,34].

Although the 50c EIA had the lowest apparent concentration of the three assays, LC-MS of the fractionated samples, together with cross-reactivity measurements, indicated that THF was the metabolite responsible for this activity. The antibody utilized in the 50c assay was raised against THF’s corticosterone counterpart, tetrahydrocorticosterone, making this cross-reactivity unsurprising. From the untargeted LC-MS analysis, THF was the most abundant metabolite that we were able to identify. Thus, taken together, these data suggest that THF, and hence the 50c EIA, are likely the best candidate metabolite and EIA, respectively, for the measurement of increased adrenocortical activity in koalas. Importantly, this metabolite was detected in both animals, suggesting it may be a more robust marker of circulating cortisol. In contrast, the cortisol and 37e EIAs showed responses against 3β-allo-THF, which was only detected in the male sample. This illustrates how the use of an EIA may provide divergent results due to variation in the FCM profile of different animals and highlights the importance of focusing on metabolites that are consistently detected.

The results here are intriguing, despite the small sample size and the potential for artifacts arising from the intravenous injection of HCSS. However, recent research [35] on a larger number of koalas, has biologically validated these three EIAs, and found 50c to be the most suitable at evaluating adrenocortical activity in this species.

Another important aspect of this study is the delay time of FCMs to be excreted and detected by LC-MS and the cortisol, 37e, and 50c EIAs after the injection. The male and female’s pellets showed a sharp increase in FCM values at around 10 h after the HCSS IV injection. Other studies validating cortisol EIAs, using an ACTH challenge, detected different timing of FCM excretion. One study [32] detected FCM elevations after 24–48 h. Blood tests measuring plasma cortisol concentration were taken 15 min post ACTH intramuscular injection; therefore, it is not clear when the exact time of the highest peak of plasma cortisol elevation might have been within the initial 15 min. Therefore, even considering a delay in elevation of plasma cortisol, due to the physiological response time to the ACTH injection, it is interesting to note that the increase in FCM values were detected several hours later than in our study. Moreover, while we collected every defecation before and after the IV injection, in the previously mentioned study [32], faeces were collected infrequently (daily), hence the actual peak time is not known.

A similar study [17] also found values of FCM increased 36 h after ACTH challenge with faecal samples collected once daily for 7 days. Since we injected cortisol intravenously, our assumption is that this is the reason for the shorter time it took to detect an increase in FCM values. The increase of FCM values detected with cortisol EIAs, in the above mentioned studies, were higher in females than in males after the ACTH challenge [17,32]. In our study the higher FCM values for the female were only detected with the cortisol EIA while values measured by 37e EIA were higher for the male, but they were similar for both sexes with 50c EIA (Figure 3).

## 4. Materials and Methods

### 4.1. Koalas

**Figure 5:**
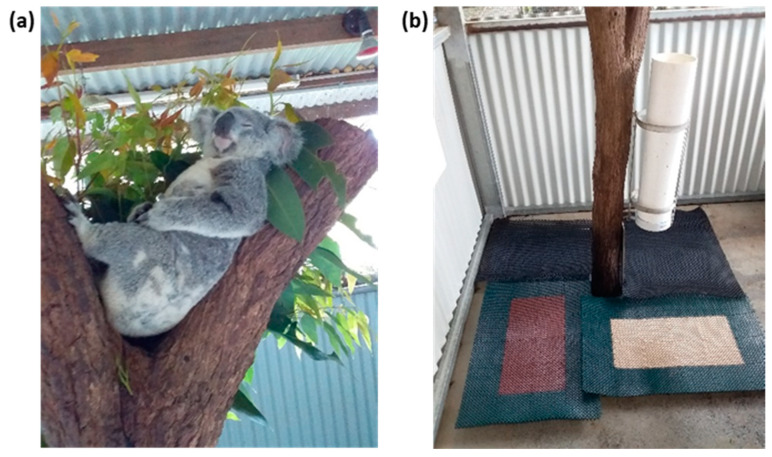
(**a**) Koala Harvey and mats used for faeces collection (**b**).

One female (Pixie, 18 months old, 4.1 kg) and one male (Harvey, 4 years old, 6.95 kg) koala housed in their usual enclosure at Kuranda Koala Gardens Kuranda, Queensland, were included in the trial. Both koalas were born and raised in captivity. They were selected from a colony of around 15, because of their placid nature (Figure 5a) and because they were used to being handled by the public. Both koalas were clinically healthy, as assessed through regular health checks. The two koalas were housed separately in adjacent and roofed outdoor enclosures. These enclosures were not accessible by the public. Food and water were provided to both koalas ad libitum. Prior to the trial, the health of each koala was assessed by Kuranda Koala Gardens’ veterinarian Dr. Peter Barratt (B.V.M.S.). Clinical examination and a blood profile showed that the two koalas were in good health.

### 4.2. Trial Design and Collection of Faecal Samples

Cortisol injection was performed by the intravenous administration of hydrocortisone sodium succinate (Solu Cortef^®^-Pfizer) at a dose of 5 mg/kg weight (Pixie, 21 mg and Harvey, 35 mg) by the veterinarian at 07:22 (Pixie) and at 07:30 (Harvey). During drug administration, the koalas were held by the koala Keeper.

Koalas were observed both day and night during the project to ensure the welfare of the animals and to record the precise time of defecation (see Supplementary Materials Table S2). Faecal samples were collected inside the koala enclosures at the base of tree stumps. Trays were placed around the tree stumps, covered by rubber hollow mats to separate faeces from urine (Figure 5b). All voided faeces were collected immediately following each defecation over a 96 h period, starting 24 h before (BC) and ending 72 h after (AC) intravenous injection of cortisol, with the time of the HCSS injection being the starting of Day 1 AC.

Pellets were stored immediately following collection and transported at −20 °C and stored at −80 °C upon return to the laboratory. Samples were initially shipped on dry ice to the Monash Proteomics and Metabolomics Facility (MPMF; Melbourne, VIC, Australia) for LC-MS analysis.

### 4.3. Sample Preparation

Multiple pellets from each defecation were ground while frozen using a mortar and pestle into a fine powder. Samples were extracted by the addition of 10 volumes (e.g., 10 µL per mg of ground faeces) in 80% methanol *v*/*v* followed by mixing for 30 min using either an orbital shaker or a vibrating mixer before centrifugation to pellet the solid material before transfering the supernatant (extract) to a clean vial.

For EIA analysis, the solvent from a 250 µL aliquot was evaporated to dryness before being shipped from Central Queensland University (Rockhampton, QLD, Australia) to the University of Veterinary Medicine (Vienna, Austria) for analysis.

For untargeted LC-MS analysis, the extracts were stored at −80 °C until immediately prior to analysis at which point they were again centrifuged (21,130× *g*, 4 °C for 30 min) and then transferred to an LC-MS vial for analysis.

### 4.4. EIA Analysis

Following analyses at MPMF, the remaining samples were shipped on dry ice to Central Queensland University. Pellets from all samples were extracted and the dried extract was sent to the University of Veterinary Medicine for EIA analyses.

Here, we utilized five EIAs that have been described previously as indicated in Table 4. Briefly, dried sample extracts were resolubilized in 80% methanol and then diluted 10-fold by the addition of assay buffer. Similarly, HPLC fractions were reconstituted in 80% methanol so as to match their volume prior to being dried down for shipping before being further diluted with assay buffer for analysis. Please note that EIAs yield relative FCM concentrations because groups of metabolites with different cross-reactivity are detected [14].

### 4.5. Standards

The six standards in Table 2 were purchased from Steraloids, Inc. Newport, RI, USA. Hydrocortisone-21-hemisuccinate sodium salt (for HPLC-MS analysis) was purchased from Sigma-Aldrich, Brisbane, Australia.

### 4.6. HPLC Fractionation of Pooled Samples

Samples were extracted and then further purified using solid phase extraction (SPE) (Oasis HLB, 3 mL, 60 mg, Waters, Rydalmere, Australia) according to the manufacturer’s instructions. Briefly, cartridges were preconditioned with 5 mL of methanol and then 5 mL of water. Samples extracted in 80% methanol as described above (Section 4.3) were diluted by the addition of water to give a final methanol concentration of 10%. These were loaded onto the cartridge and the flow through was discarded. The sample was then eluted in 1.8 mL of 90% methanol and the solvent removed under vacuum. Each sample was then reconstituted in 100 µL 90% methanol and combined with other samples as appropriate to provide pooled samples for before and after cortisol administration for each animal.

Then, 100 µL of the pooled sample was dried under a stream of nitrogen gas and reconstituted in 100 µL of 10% acetonitrile. Next, 40 µL of this sample was then fractionated on a 1260 Infinity II (Agilent, Melbourne, VIC, Australia) HPLC by reverse-phase chromatography using a Zorbax 300Extend-C18 4.6 × 250 mm 5 micron (Agilent) column with a water acetonitrile gradient with 0.1% formic acid. The gradient ran from 15% to 40% acetonitrile over 50 min, then up to 100% acetonitrile over the next 2 min, remained there for 5 min before re-establishing the original conditions and re-equilibrating for the next run. Fractions were collected in 60 s slices except for between 22 to 37 min where they were collected in 30 s slices. The flow rate was maintained at 500 µL/min. Finally, 100 µ of each fraction was reserved for LC-MS analysis while the remainder was dried under vacuum and shipped from Monash University (Melbourne, VIC, Australia) to the University of Veterinary Medicine (Austria) for EIA analysis.

### 4.7. HPLC-MS Analysis

Unless otherwise specified, LC-MS experiments were performed on a Dionex RSLC3000 UHPLC coupled to a Q-Exactive Orbitrap MS (Thermo Fisher Scientific, Melbourne, VIC, Australia). For the untargeted metabolomics portion of the study, samples were analysed by hydrophilic interaction liquid chromatography (HILIC) following a previously published method [40,41]. In brief, the chromatography utilized a ZIC-p(HILIC) column 5 µm 150 × 4.6 mm with a 20 × 2.1 mm ZIC-pHILIC guard column (both Merck Millipore, Melbourne, VIC, Australia) at 25 °C. A gradient elution of 20 mM ammonium carbonate (A) and acetonitrile (B) (linear gradient time, %B as follows: 0 min, 80%; 15 min, 50%; 18 min, 5%; 21 min, 5%; 24 min, 80%; 32 min, 80%) was utilized. The flow rate was maintained at 300 μL/min. Samples were kept at 6 °C in the autosampler and 10 μL was injected for analysis. The mass spectrometry was performed at 35,000 resolutions, operating in rapid switching positive (4 kV) and negative (−3.5 kV) modes for electrospray ionization (capillary temperature 300 °C; sheath gas 50; Aux gas 20; sweep gas 2; probe temp 120 °C). Similarly, Reverse phase LC-MS was performed using the same LC-MS instrumentation described above using an Accucore C18 150 × 2.1 mm, 2.6 µm (Thermo Fisher Scientific) column. A gradient elution of 10 mM ammonium formate (A) and acetonitrile (B) was utilized (linear gradient time, %B: 0 min, 15%; 15 min, 50%; 20 min, 100%; 25 min, 100%; 26 min, 15%; 31 min 15%). The flow rate was 400 µL min^−1^ and the column was operated at 40 °C.

Additionally, targeted LC-MS analysis was performed using a 1290 Infinity II HPLC and 6495 Triple Quadrupole mass spectrometer (Agilent) operating in multiple reaction monitoring (MRM) mode. The transitions and conditions are included in Supplementary Materials Table S2. Chromatography was conducted using a Zorbax Eclipse Plus C18 2.1 × 100 mm, 1.8 µm in series with a matching 5 mm guard column (Agilent). A gradient elution of 0.1% formic acid (A) and acetonitrile with 0.1% formic acid (B) (linear gradient time, %B as follows: 0 min, 0%; 15 min, 100%; 19 min, 100%; 21 min, 0%; 26 min, 0%) was utilized with a flow rate of 300 µL/min.

### 4.8. Processing of the Untargeted HILIC LC-MS Data

The LC-MS data were processed by applying the first three steps of the IDEOM v20 [42] workflow. Specifically, data corresponding to each polarity were extracted to separate files in mzXML format using MSConvert (http://proteowizard.sourceforge.net/, accessed on 16 June 2021). Untargeted extraction of chromatographic features (corresponding to a specific mass) and alignment was then performed using the XCMS algorithm [43] and mzMatch, respectively, implemented through R [44]. This resulted in two data matrices, corresponding to each polarity, which were exported into excel for further analysis to identify features that were elevated following the administration of hydrocortisone sodium succinate. Samples from the two animals were analysed independently.

### 4.9. Ethics

This project was completed in accordance with the Central Queensland University’s Animal Ethics Committee number 0000020593 and the Queensland Department of Environment and Science Scientific Purpose Permit WISP18537317.

## 5. Conclusions

In this study we utilized both LC-MS and several EIAs to identify the major metabolites of cortisol in the faeces of koalas. LC-MS analysis identified THF along with several of its isomers as FCMs. Similarly, following a survey of five EIAs, we found that three gave a substantial response to FCMs. The cortisol and 37e EIAs both responded to 3β-allo-THF while the 50c EIA responded to THF. THF was detected in samples from both animals studied and of the metabolites identified gave the most abundant signal by LC-MS. In contrast, 3β-allo-THF was detected in only one of the two animals studied. Taken together, these results suggest that THF is likely to be the main FCM in koalas and, accordingly, 50c is the most promising EIA for the non-invasive assessment of stress in koalas.

## Figures and Tables

**Table 1:** Chromatographic features detected in the untargeted LC-MS analysis that were elevated in the samples following administration of cortisol.

Feature	Mass (Da)	RT (min)	Polarity	Koalas	Comment
1	462.22579	7.12	Pos	Harvey	Consistent with hydrocortisone succinate (mass = 462.22537, MF = C_25_H_34_O_8_)
	462.22484	7.25	Pos	Pixie	
2	366.24031	6.28	Neg	Harvey	Consistent with tetrahydrocortisol or isomers (mass = 366.24063, MF = C_21_H_34_O_5_)
	366.23961	5.38	Neg	Pixie	
3	382.23521	7.35	Neg	Harvey	Related to feature 2 by addition of an oxygen (mass = 382.23554, MF = C_21_H_34_O_6_)
	382.23475	7.43	Neg	Pixie	
4	384.25102	7.46	Neg	Harvey	Related to feature 2 by addition of water (mass = 384.25119), MF = C_21_H_36_O_6_)
	384.25048	7.53	Neg	Pixie	
5	352.22484	7.46	Neg	Harvey	Related to feature 2 by the loss of CH_2_ (mass = 352.22497, MF = C_20_H_32_O_5_)
	352.22445	7.47	Neg	Pixie	
6	483.28366	4.35	Pos	Harvey	Consistent with the ammonium adduct of tetrahydrocortisol succinate (or isomer) (mass = 483.28322, MF = C_25_H_41_NO_8_)
	483.28373	4.26	Pos	Pixie	

**Table 2:** Details of the standards of potential cortisol metabolites.

Abbreviation	Standard	Trivial Name
THF	5β-pregnan-3α,11β,17α,21-tetrol-20-one	tetrahydrocortisol
allo-THF	5α-pregnan-3α,11β,17α, 21-tetrol-20-one	allotetrahydrocortisol
3β-THF	5β-pregnan-3β,11β,17α,21-tetrol-20-one	3β-tetrahydrocortisol
3β-allo-THF	5α-pregnan-3β,11β,17α,21-tetrol-20-one	3β-allotetrahydrocortisol
α-cortolone	5β-pregnan-3α,17α,20α,21-tetrol-11-one	α-cortolone
β-cortolone	5β-pregnan-3α,17α,20β,21-tetrol-11-one	β-cortolone

**Table 3:** Percentage cross-reactivity of several steroids with the three different EIAs. Cross-reactivity is relative to the respective standards (cortisol, tetrahydrocorticosterone for 50c, and 3β,5α-tetrahydrocorticosterone for 37e).

Compound	Cortisol	50c	37e EIAs
THF	<0.001	20.7	<0.001
Allo-THF	1.4	<0.01	0.5
3β-THF	0.1	<0.001	0.2
3β-allo-THF	25.0	<0.001	100.0
α-cortolone	<0.0001	<0.01	0.1
β-cortolone	<0.0001	<0.01	<0.001

**Table 4:** Details of the EIAs utilized in the current study.

EIA Code	Details	Description
69a	Standard	5β-androstane-3α,11β-diol-17-one (11β-hydroxyaetiocholanolone)
	Targeted structure	5β-3α,11β-diol
	Antibody against	5β-androstane-3α,11β-diol-17-one-CMO:BSA
	Label	5β-androstane-3α,11β-diol-17-one-CMO-biotinyl-LC
	Reference	Frigerio et al. [36]
72T	Standard	5β-androstane-3α-ol-11,17-dione (11-oxoaetiocholanolone)
	Targeted structure	5β-3α-ol-11-one
	Antibody against	5β-androstane-3α-ol-11,17-dione-CMO:BSA
	Label	5β-androstane-3α-ol-11,17-dione-CMO-biotinyl-3,6,9-trioxaundecanediamin
	Reference	Möstl et al. [13]
Cortisol	Standard	4-pregnene-11β,17α,21-triol-3,20-dione(cortisol)
	Targeted structure	11β,17α,21-triol-20-one
	Antibody against	cortisol-3-CMO:BSA
	Label	cortisol-3-CMO-DADOO-biotin
	Reference	Palme and Möstl [37]
37e	Standard	5α-pregnane-3β,11β,21-triol-20-one(3β-allotetrahydrocorticosterone)
	Targeted structure	5α-3ß,11ß-diol
	Antibody against	5α-pregnane-3β,11β,21-triol-20-one-CMO
	Label	5α-pregnane-3β,11β,21-triol-20-one-CMO-biotinyl-LC
	Reference	Touma et al. [38]
50c	Standard	5β-pregnane-3α,11β,21-triol-20-one(tetrahydrocorticosterone)
	Targeted structure	5β-3α,11β-diol
	Antibody against	5β-pregnane-3α,11β,21-triol-20-one-CMO
	Label	5β-pregnane-3α,11β,21-triol-20-one-21-HS-biotinyl-LC
	Reference	Quillfeldt and Möstl [39]

## Data Availability

Data from the untargeted LC-MS analysis is publicly available at https://store.erc.monash.edu/experiment/view/13694/, accessed on 16 June 2021.

## Supplementary Materials

The following are available online at https://www.mdpi.com/article/10.3390/metabo11060393/s1, Figure S1: MS/MS spectra of parent ion 411.2388 in the RT region around 9.1 (a) and 8.3 (b) minutes for a representative AC sample extract (The male 5th AC sample) top and THF (a) and α-Cortolone (b) standards below. The fragmentation spectra of the standards closely match those for the standards, Figure S2: Extracted ion chromatograms (EIC) corresponding to deprotonated hydrocortisone succinate for the 13th AC female sample (a) and an authentic standard (b), Figure S3: (a) and (b) show candidate structures consistent with the mass of feature 6 corresponding to succinate esters of THF and cortolone respectively. EIC corresponding to deprotonated feature 6 for the 4th AC sample from the male (c) and 9th AC sample from the female (d), Figure S4: EIAs used for analyses of faecal extracts. (a) 72t and 69a EIA. Red lines represent Pixie and black Harvey, Table S1: MRM conditions used on triple quadrupole mass spectrometer, Table S2: Details of the faecal sample collection.
