# Supplementary material for: Identification of Koala (Phascolarctos cinereus) Faecal Cortisol Metabolites Using Liquid Chromatography-Mass Spectrometry and Enzyme Immunoassays
Source: Metabolites. 2021 Jun 16;11(6):393. doi: 10.3390/metabo11060393 (PMC8234238; doi:10.3390/metabo11060393)
Supplement: Supplementary file 1 [file metabolites-11-00393-s001.zip › metabolites-1253189-supplementary.pdf]

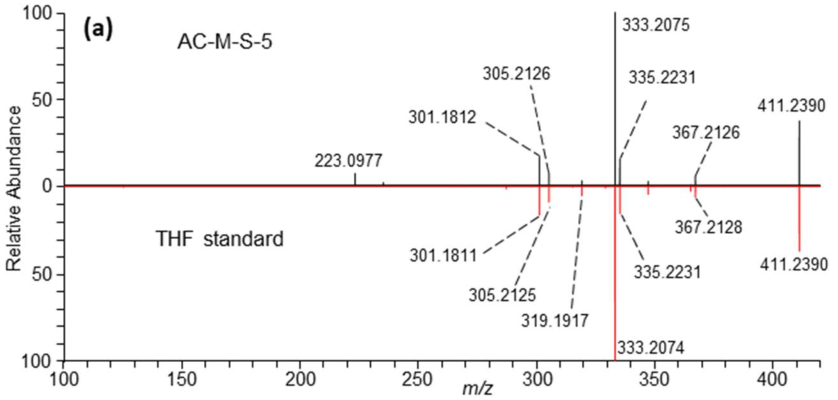

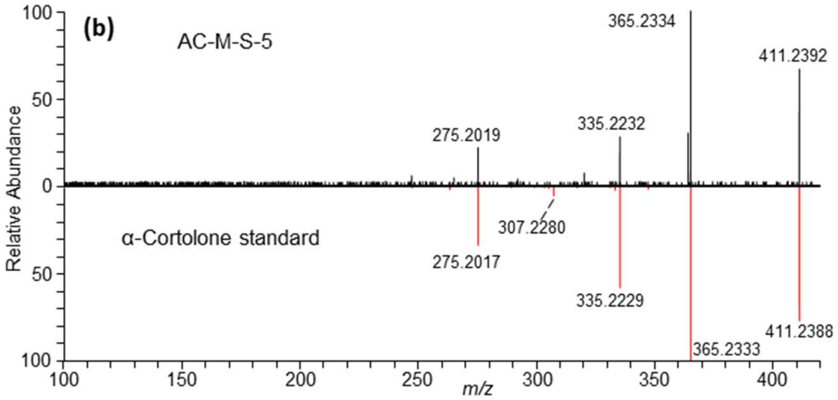

Figure S1: MS/MS spectra of parent ion 411.2388 in the RT region around 9.1 (**a**) and 8.3 (**b**) minutes for a representative AC sample extract (The male 5th AC sample) top and THF (**a**) and α-Cortolone (**b**) standards below. The fragmentation spectra of the standards closely match those for the standards.

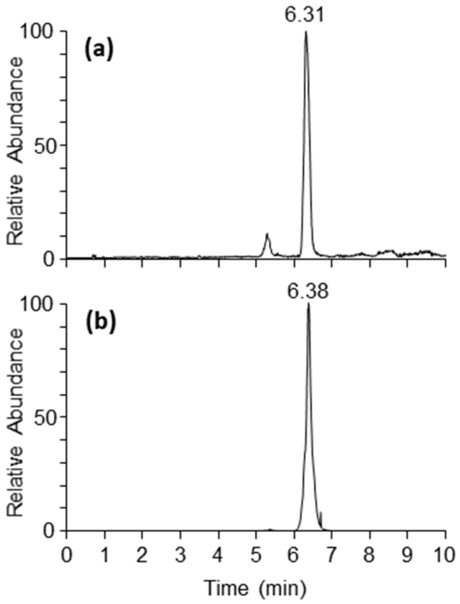

Figure S2: Extracted ion chromatograms (EIC) corresponding to deprotonated hydrocortisone succinate for the 13th AC female sample (**a**) and an authentic standard (**b**).

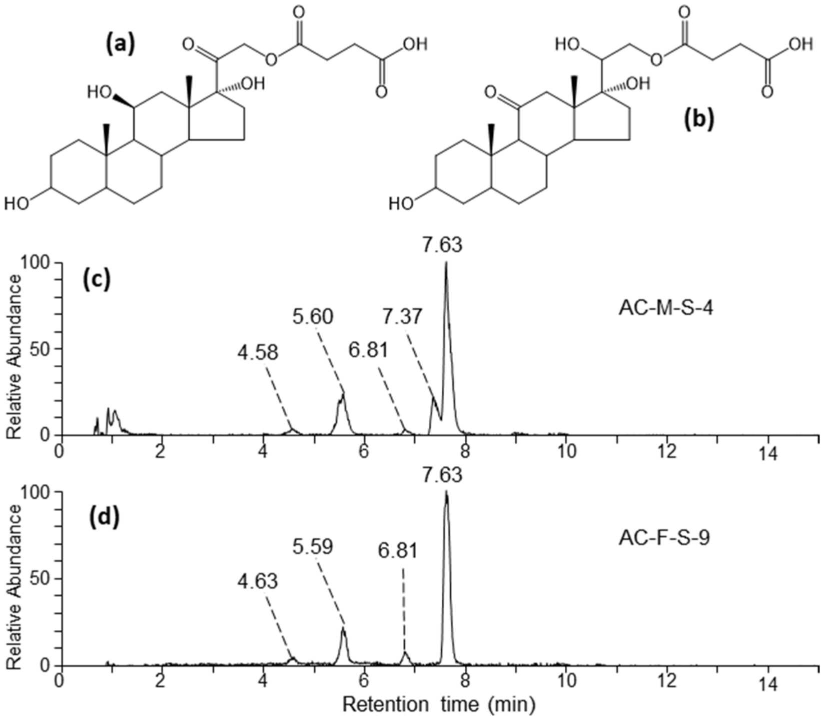

Figure S3: (a) and **(b)** show candidate structures consistent with the mass of feature 6 corresponding to succinate esters of THF and cortolone respectively. EIC corresponding to deprotonated feature 6 for the 4th AC sample from the male (**c**) and 9th AC sample from the female (**d**).

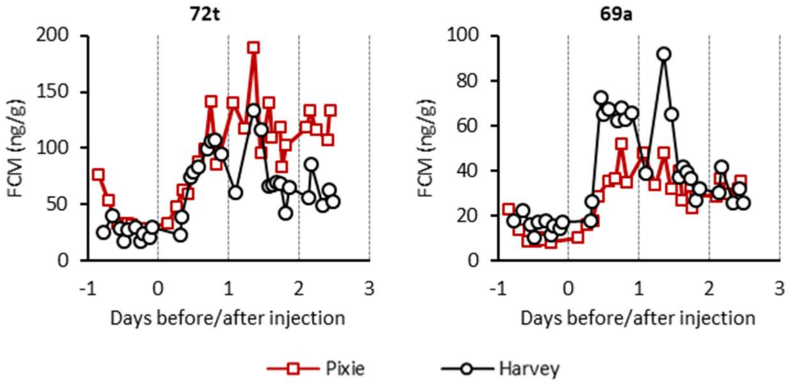

Figure S4: EIAs used for analyses of faecal extracts. (a) 72t and 69a EIA. Red lines represent Pixie and black Harvey.

Table S1: MRM conditions used on triple quadrupole mass spectrometer. Note that the same transitions were used for all the tetrahydrocortisol isomers.

Analyte(s)	Transition	Polarity	Collision Energy
Cortisol	362.2 → 121.0	negative	26
Cortisol	362.2 → 267.1	negative	18
Cortisol	362.2 → 309.1	negative	16
Cortisol	362.2 → 327.1	negative	14
Tetrahydrocortisol isomers	331.1 → 295.1	positive	12
Tetrahydrocortisol isomers	331.1 → 301.1	positive	8
Tetrahydrocortisol isomers	331.1 → 313.1	positive	10
Tetrahydrocortisol isomers	349.1 → 295.1	positive	14
Tetrahydrocortisol isomers	349.1 → 301.1	positive	12
Tetrahydrocortisol isomers	349.1 → 313.1	positive	12
Tetrahydrocortisol isomers	411.3 → 313.1	negative	18
11-oxoaetiocholanolone	287.1 → 229.1	positive	22
11-oxoaetiocholanolone	287.1 → 269.1	positive	18

Table S2: Details of the faecal sample collection.

	Animal	Day	Date	Time	Number of pellets
Before cortisol administration	Harvey	1	25/09/2018	12:55-13:05	31
		1	25/09/2018	16:00-16:19	53
		1	25/09/2018	18:30-18:34	23
		1	25/09/2018	19:55-20:02	13
		1	25/09/2018	21:00-21:45	45
		1	25/09/2018	22:35-22:43	23
		1	25/09/2018	23:56	30
		1	26/09/2018	01:38-01:43	25
		1	26/09/2018	02:39-02:48	15
		1	26/09/2018	04:44-04:55	19
After cortisol administration	Harvey	1	26/09/2018	05:25-05:35	22
		1	26/09/2018	14:59-15:04	20
		1	26/09/2018	15:30-15:40	26
		1	26/09/2018	18:25-18:37	19
		1	26/09/2018	19:20-19:30	10
		1	26/09/2018	21:13-21:26	32
		1	27/09/2018	00:07-00:26	47
		1	27/09/2018	01:27-02:30	32
		1	27/09/2018	3:04	9
		1	27/09/2018	4:56	43
		2	27/09/2018	09:45-09:50	10
		2	27/09/2018	16:00-16:12	32
		2	27/09/2018	18:40-19:15	38
		2	27/09/2018	21:05-21:18	23
		2	27/09/2018	22:30-23:06	29
		2	28/09/2018	00:02-0050	34
		2	28/09/2018	01:13-01:34	18
		2	28/09/2018	3:00	33
		2	28/09/2018	4:10-4:20	6
		3	28/09/2018	10:36-10:45	24
		3	28/09/2018	11:43-12:00	30+
		3	28/09/2018	15:35-16:00	30
		3	28/09/2018	17:35-17:52	37
		3	28/09/2018	19:05-19:15	10

Table S3 continues on the following page

Table S2 continued

	Animal	Day	Date	Time	Number of pellets
Before cortisol administration	Pixie	1	25/09/2018	11:06-11;25	25
	Pixie	1	25/09/2018	14:21-14:30	22
	Pixie	1	25/09/2018	17:30-17:34	24
	Pixie	1	25/09/2018	20:10-20:15	10
	Pixie	1	25/09/2018	21:05-21:30	12
	Pixie	1	25/09/2018	22:22-22:29	8
	Pixie	1	26/09/2018	01:22-01:28	13
	Pixie	1	26/09/2018	03:07-03:10	3
	Pixie	1	26/09/2018	04:30-04:32	2
After cortisol administration	Pixie	1	26/09/2018	10:36-10:42	23
	Pixie	1	26/09/2018	13:54-14:05	23
	Pixie	1	26/09/2018	15:52-16:09	16
	Pixie	1	26/09/2018	17:27-18:13	17
	Pixie	1	26/09/2018	18:25-18:45	3
	Pixie	1	26/09/2018	19:06-19:39	7
	Pixie	1	26/09/2018	20:55	9
	Pixie	1	26/09/2018	23:29-23:35	6
	Pixie	1	27/09/2018	01:27-01:40	9
	Pixie	1	27/09/2018	3:16	13
	Pixie	1	27/09/2018	5:14	11
	Pixie	2	27/09/2018	09:00-09:15	29
	Pixie	2	27/09/2018	12:57-13:10	33
	Pixie	2	27/09/2018	unknown	drier pellets
	Pixie	2	27/09/2018	18:22-19:15	15
	Pixie	2	27/09/2018	21:15-21:28	18
	Pixie	2	28/09/2018	22:08-22:23	27
	Pixie	2	28/09/2018	01:02-00:35	23
	Pixie	2	28/09/2018	01:39-02:00	21
	Pixie	2	28/09/2018	3:00	6
	Pixie	3	28/09/2018	9:50-10:15	38
	Pixie	3	28/09/2018	11:10-11:20	23
	Pixie	3	28/09/2018	13:15-13:20	6
	Pixie	3	28/09/2018	17:00-17:15	37
	Pixie	3	28/09/2018	17:58-18:06	8
